# Contrasting population structure and demographic history of cereal aphids in different environmental and agricultural landscapes

**DOI:** 10.1002/ece3.6565

**Published:** 2020-09-11

**Authors:** Ramiro Morales‐Hojas, Jingxuan Sun, Fernando Alvira Iraizoz, Xiaoling Tan, Julian Chen

**Affiliations:** ^1^ Rothamsted Insect Survey Biointeractions and Crop Protection Department Rothamsted Research Harpenden UK; ^2^ State Key Laboratory for Biology of Plant Diseases and Insect Pests Institute of Plant Protection Chinese Academy of Agricultural Sciences Beijing China; ^3^ Functional and Evolutionary Entomology Gembloux Agro‐Biotech University of Liège Gembloux Belgium

**Keywords:** cereal aphids, China, England, insecticide resistance, population genetics

## Abstract

Genetic diversity of populations has important ecological and evolutionary consequences, whose understanding is fundamental to improve the sustainability of agricultural production. Studies of how differences in agricultural management and environment influence the population structure of insect pests are central to predict outbreaks and optimize control programs. Here, we have studied the population genetic diversity and evolution of *Sitobion avenae* and *Sitobion miscanthi*, which are among the most relevant aphid pests of cereals across Europe and Asia, respectively. We have used genotyping by sequencing (GBS) to identify genome‐wide single nucleotide polymorphisms (SNPs) to infer the geographic structure and migration patterns. In the present study, we show that the population structure in present‐day populations is different from that described in previous studies, which suggest that they have evolved recently possibly as a response to human‐induced changes in agriculture. This study shows that *S. avenae* in England is predominantly parthenogenetic and there has been a demographic and spatial expansion of a single genetic cluster, which could correspond with the insecticide resistance superclone identified in previous studies. Conversely, in China, *S. miscanthi* populations are mostly cyclical parthenogenetic, with one sexual stage in autumn to produce overwintering eggs, and there are six genetically differentiated subpopulations and high genetic differentiation between geographic locations, which suggests that further taxonomical research is needed. Unlike *S. avenae* in England, there is no evidence for insecticide resistance and there is no predominance of a single lineage in *S. miscanthi* in China.

## Graphical abstract

We analyze the population genetic structure of two cereal aphid species in the UK and China. The UK shows signature of expansion of a single clone, while in China, the species is more diverse and cryptic diversity is possible.

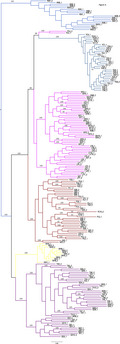

## 1 INTRODUCTION

A major challenge in agricultural entomology is to develop efficient control strategies for pest organisms. For this, it is important to understand how environmental and anthropogenic factors influence the genetic structure and the evolutionary dynamics of insect populations (Pelissie, Crossley, Cohen, & Schoville, 2018). The level of genetic structure and diversity is the result of a combination of several factors which include selection, migration, and life history (i.e., reproduction mode) (Leffler et al., 2012), and studying their consequences on insect populations is of great interest to improve ecological agricultural practices (Pelissie et al., 2018). The use of pesticides remains a necessary way to control and manage pests in agriculture. However, their use imposes a strong selection pressure on pest populations and resistance to different types of insecticides has, therefore, evolved in many insects (Bass, Denholm, Williamson, & Nauen, 2015; Georghiou, 1972). Designing new strategies of pest control that rationalize the use of insecticides and reduce the likelihood of an evolution of resistance is key for the development of sustainable agriculture practices that reduce the environmental footprint (Wijnands, 1997), and understanding how pest populations respond to selective pressure and adapt to ecological changes is key to design rational strategies of management and control that are more targeted. In addition, a better understanding of the geographic connectivity between populations and the dispersal capacity of pests provides valuable information to control their abundance and distribution, while preventing also the spread of adaptive genetic variation, such as insecticide resistance, across their geographic range (Mazzi & Dorn, 2012). Therefore, it is essential that we incorporate the fundamental knowledge about population genetics into agricultural entomology.

Aphids comprise some of the most pernicious species of crop pests. In cereals, *Sitobion avenae* and *Sitobion miscanthi* are two of the most economically important species in Europe and Asia, respectively, and they are major vectors of the barley yellow dwarf virus (BYDV), which can severely reduce cereal yield (Vickerman & Wratten, 1979). Both *Sitobion* species are monoecious, nonhost‐alternating, feeding only on Poaceae grasses and cereals (Blackman & Eastop, 2017). Like many aphids, *S. avenae* and *S. miscanthi* show different levels of variation in their life cycle, from individuals that are obligate cyclical parthenogenetic and have a generation that undergoes sexual reproduction in the primary host (holocycly), to clones that are obligate parthenogenetic and reproduce asexually all year round (anholocycly) (Dedryver, Le Gallic, Gauthier, & Simon, 1998). In addition, individuals can remain asexual in the cereal crops during winter as a response to environmental cues such as warmer temperatures and day length (Dedryver et al., 1998). As a result, a geographic cline in the reproductive type has been described in the *S. avenae* populations of UK and France, with increasing proportion of sexual reproduction toward the north of the countries (Llewellyn et al., 2003; Simon et al., 1999). In the case of *S. miscanthi*, variation in the life cycle has also been described. Populations from this species in Australia and New Zealand are anholocyclic, while they are holocyclic in Taiwan (Sunnucks, England, Taylor, & Hales, 1996; Wilson, Sunnucks, & Hales, 1999). In China, *S. miscanthi* has been traditionally reported to be anholocyclic (Guo, Shen, Li, & Gao, 2005; Zhang, 1999). However, contrary to the observations in *S. avenae* and other species, a recent population genetics study has observed signatures of cyclical parthenogenesis in the southern populations of the country while obligate parthenogenetic reproduction would be dominant in the north (Wang, Hereward, & Zhang, 2016).

Resistance to pyrethroids was first detected in the UK populations of *S. avenae* in 2011. This was due to a knockdown resistance (*kdr*) mutation (L1014F) in the sodium channel gene (Foster et al., 2014). This knockdown appeared as a heterozygous mutation in one clone of *S. avenae*, known in the literature as clone SA3, and rapidly increased its abundance in the UK population from 2009 to 2014, although in variable proportions in different locations and years (Dewar & Foster, 2017; Malloch, Foster, & Williamson, 2016; Malloch, Williamson, Foster, & Fenton, 2014). The spread of the mutation in the UK was limited by the fact that the SA3 clone is anholocyclic, so pyrethroid resistance has not spread to other lineages through sexual recombination. In addition, the high connectivity of the UK populations revealed using four microsatellite loci (Llewellyn et al., 2003), probably facilitated the geographic spread of the resistant clone from its location of origin. Therefore, the continued use of pyrethroids combined with the long dispersal capacity of *S. avenae* has likely favored the spread of this clone across the UK. Nevertheless, the clonal diversity inferred using microsatellites remained high and similar to the diversity before the evolution of pyrethroid resistance, and other susceptible clones and phenotypes were still present in different proportions in British populations by 2015 (Llewellyn et al., 2003; Malloch et al., 2016). In the case of *S. miscanthi*, there is no available information in the literature regarding the evolution of insecticide resistance. However, understanding the dynamics and movement of the species can help manage and control the damage in cereal crops, and establish management programs to reduce the likelihood of insecticide resistance evolution. In China, previous studies have shown high levels of genetic diversity in *S. miscanthi* using a panel of five microsatellites (Guo et al., 2005; Wang et al., 2016), similar to those reported for *S. avenae* in the UK and France, and there is genetic differentiation between north and south of the country but low differentiation within each region, suggesting free gene flow within geographic regions (Guo et al., 2005; Wang et al., 2016).

In the present study, we analyze the population genetics and demographic history of *S. avenae* and *S. miscanthi* in England and China, respectively, using genotyping by sequencing (GBS) to identify potential weak differentiation. We discuss the results in view of the differences in life‐history types and the evolution of insecticide resistance, which may be limited by the reproductive type. These genomics approaches have identified genetic variation at a national and regional scale for other aphids in regions where they disperse long distances (Morales‐Hojas et al., 2019).

## 2 MATERIALS AND METHODS

### 2.1 Samples

**FIGURE 1:**
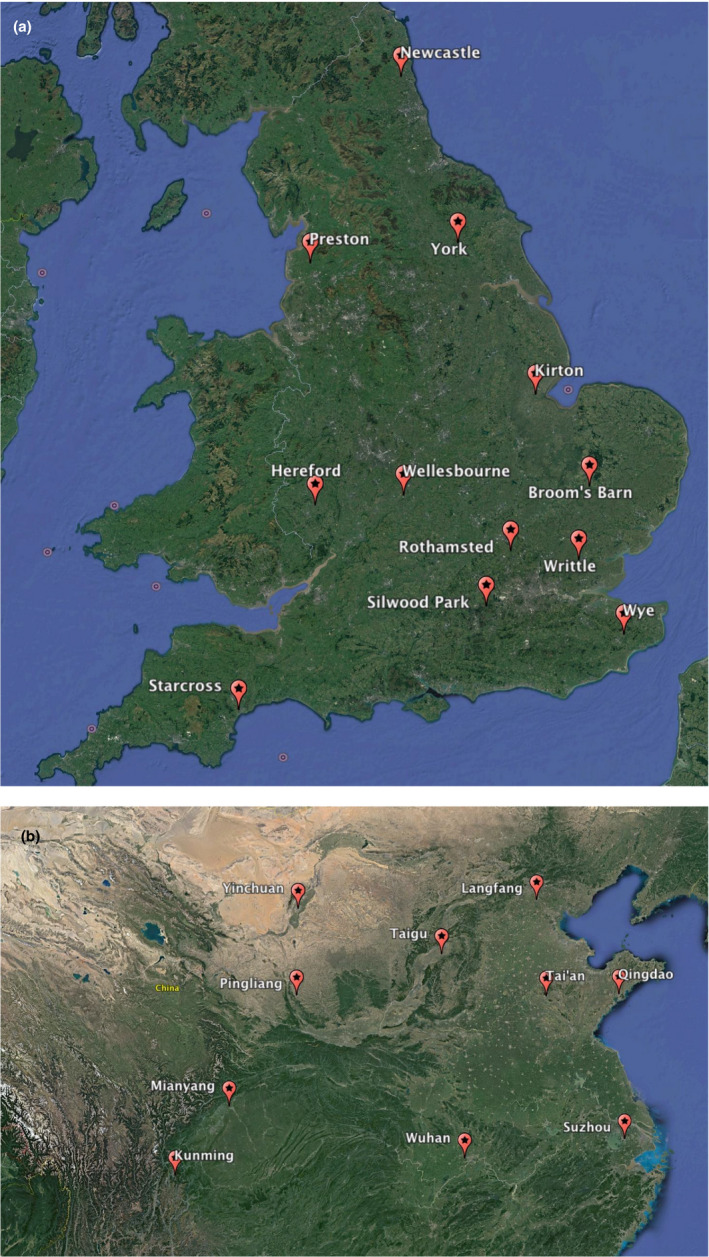
Maps showing the locations where samples of *S. avenae* were collected in England (a) and where *S. miscanthi* aphids were collected in China (b)

Individuals of *S. avenae* were collected using the network of 12.2 m high suction traps that is run by Rothamsted Insect Survey (RIS). The RIS suction traps are continuously collecting flying insects, and during the aphid season, aphid samples are identified daily to species level (Morales‐Hojas, 2017; Storkey et al., 2016); of the identified aphids, 10 individuals of *S. avenae* collected during June–July 2018 with suction traps located in 12 sites across England (Starcross, Wye, Writtle, Broom's Barn, Kirton, Rothamsted, Silwood Park, Wellesbourne, Hereford, Preston, York, and Newcastle; see Table 1 and Figure 1) were used for this study. Individuals of *S. miscanthi* were collected in 10 sites (Kunming, Mianyang, Wuhan, Qingdao, Tai'an, Pingliang, Yinchuan, Langfang, Taigu, and Suzhou) across the cereal growing areas of China between February and June of 2017 (Table 1, Figure 1). The 10 individuals of *S. miscanthi* were collected from the same wheat field but from plants separated by 10 m to reduce the probability of sampling the same clone.

**TABLE 1:** Locations and number of samples (*N*) used in the present study

Country	Location	Geographic coordinates	*N*
UK	Broom's Barn (BB)	52.260681, 0.56843	10
UK	Hereford (H)	52.124201, −2.638156	10
UK	Kirton (K)	52.924454, −0.052153	10
UK	Newcastle (N)	55.213254, −1.685083	10
UK	Preston (P)	53.854383, −2.76699	10
UK	Rothamsted (RT)	51.806997, −0.360091	10
UK	Silwood Park (SP)	51.40941, −0.643357	10
UK	Starcross (SX)	50.629596, −3.45463	10
UK	Wellesbourne (We)	52.205975, −1.605017	10
UK	Writtle (Wr)	51.733599, 0.429233	10
UK	Wye (W)	51.185507, 0.944941	10
UK	York (Y)	54.014616, −0.97320532	10
China	Kunming (KM)	24.8855, 102.8215	10
China	Mianyang (MY)	31.5347, 104.5676	10
China	Wuhan (WH)	30.5820, 114.0292	10
China	Qingdao (QD)	36.3074, 120.3963	10
China	Tai'an (TA)	36.1920, 117.1353	10
China	Pingliang (PL)	35.5426, 106.6748	10
China	Yinchuan (YC)	38.4731, 106.2428	10
China	Langfang (LF)	39.5031, 116.6857	10
China	Taigu (TG)	37.4212, 112.5513	10
China	Suzhou (SZ)	31.3023, 120.6313	10

### 2.2 DNA extraction and SNP genotyping

DNA was extracted from samples using Qiagen's DNeasy Blood and Tissue kit following the manufacturer's protocol. Identification of SNP loci was done using GBS. Library preparation and sequencing of samples were outsourced commercially to Novogene LTD in the case of *S. avenae* and Allwegene Technology LTD *S. miscanthi*. Briefly, genomic DNA was digested with *MseI* in the case of *S. avenae* and with *ApeKI* in the case of *S. miscanthi*. The library preparation was performed following the standard Illumina pair‐end (PE) protocol, and PE sequencing of 150 bp was performed on an Illumina HiSeq platform. Read quality was assessed with FastQC v0.67, and in the case of *S. miscanthi*, the first 10 bases were trimmed due to low quality using trimmomatic 0.36.1 (Bolger, Lohse, & Usadel, 2014). Reads were mapped to a draft of the *S. avenae* genome using BWA‐MEM 0.7.16.0 letting BWA choosing the best algorithm to construct the index and the option of setting read group information Picard style. Duplicates were removed using MarkDuplicates v2.7.1.1, and indels were realigned with BamLeftAlign v1.0.2.29‐1. Variant calling was carried out with FreeBayes v1.0.2.29‐3 (Garrison & Marth, 2012) with a simple diploid calling of variants (standard filters of minimum mapping quality of 30, minimum base quality of 30, default minimum supporting allele qsum, and genotype variant threshold) and minimum coverage of 2. The resulting SNPs from FreeBayes were annotated using snpEff v4.0. These tools were run using Galaxy v17.05 (Afgan et al., 2016) and parameters provided refer to the Galaxy options. SNPs called with FreeBayes were filtered using VCFtools v0.1.14 (Danecek et al., 2011) before the markers were used in subsequent analyses. Different filtering schemes were tested in each species to obtain a dataset that maximized the quality of the SNPs and genotypes while minimizing the missing data at marker and individual levels, as recommended by O'Leary, Puritz, Willis, Hollenbeck, and Portnoy (2018). Given that GBS was performed separately for the two species, the best filtering schemes were different (vcftools parameters *S.miscanthi*: max‐missing 0.75, minDP 3, mac 3, minQ 30, remove‐indels, thin 2000, max‐missing 0.9, thin 5,000; *S. avenae*: max‐missing 0.5, mac 3, minQ 30, minDP 3, max‐missing 0.5, exclude individuals with 50% missing data, max‐missing 0.75, remove‐indels, thin 2000).

### 2.3 Analyses of population structure

The population structure of both species was investigated using the Bayesian genetic clustering algorithm implemented in Structure 2.3.4 (Pritchard, Stephens, & Donnelly, 2000). We used the admixture model with correlated frequencies, and to detect any potential subtle genetic structure, we ran Structure with the sampling locations set as priors (locprior = 1); this model has the power to detect a weak structure signal and does not bias the results toward detecting genetic structure when there is none. Analyses for the two species were performed separately and with different parameters as the datasets were obtained following different protocols (different sequencing companies) and differed in number of individuals, markers, and quality. In the case of *S. miscanthi*, a first run of Bayesian clustering analyses of the population structure was carried out with five independent simulations with 100,000 burn‐in and 100,000 mcmc chains for each of *K* 1–10. An additional run of five independent simulations with 100,000 burn‐in and 500,000 mcmc chains was carried out for *K* 5–10 to confirm the results of the first run and ensure convergence in the mcmc step. In the analyses of *S. avenae* from the UK, Structure was run with 5 replicates of 500,000 burn‐in and 1,000,000 mcmc chains for *K* ranging from 1 to 12. Summary statistics (alpha and likelihood parameters) convergence was inspected visually to confirm that the burn‐in and run lengths were adequate. We ran the Structure simulations using a multicore computer with the R package ParallelStructure (Besnier & Glover, 2013) in the CIPRES science gateway server (Miller, Pfeiffer, & Schwartz, 2010). The number of *K* groups that best fitted the dataset was estimated using the method of Evanno, Regnaut, and Goudet (2005) using Structure Harvester Web v0.6.94 (Earl & Vonholdt, 2012). Cluster assignment probabilities were estimated using the program Clumpp (Jakobsson & Rosenberg, 2007) as implemented in the webserver CLUMPAK (Kopelman, Mayzel, Jakobsson, Rosenberg, & Mayrose, 2015). To validate the identified number of clusters identified by Structure, the nonmodel‐based method of discriminant analysis of principal components (DAPC) was performed as implemented in the R package adegenet 2.1.2 (Jombart, 2008; Jombart & Ahmed, 2011; Jombart, Devillard, & Balloux, 2010). The number of genetic clusters was identified using the Bayesian information criterion (BIC) with the “find.clusters” function in adegenet 2.1.2 with manual and automatic selection (choose.n.clust = FALSE) with the good fit criterion to choose *K*.

The genetic diversity measures of the populations were estimated using Arlequin 3.5.2.2 (Excoffier, Laval, & Schneider, 2005); Fis was also estimated and tested for using the exact probability test in the R version of Genepop v. 4.7.5 (Rousset, 2008). Genetic variation among populations was investigated using an analysis of the molecular variance (AMOVA) with 10,000 permutations using Arlequin 3.5.2.2. We used hierarchical AMOVA to test the population structures resulting from the Structure runs. Population pairwise divergence was investigated using F_ST_, and the significance was evaluated with 10,000 permutations in Arlequin. We ran Mantel tests as performed in Arlequin to evaluate the correlation between the genetic distances (F_ST_) and the geographic distance between sampling locations. The geographic distances (in Km) were estimated using Google maps (Tables 5 and 10). The demographic history was also explored using Arlequin. For this, we first estimated the gametic phase from the multilocus diploid data using the ELB algorithm with the default parameter values in Arlequin 3.5.2.2 (Excoffier, Laval, & Balding, 2003). Population expansion or bottleneck was inferred using Fu's F_S_ (Fu, 1997), and mismatch analyses were run with 1,000 bootstrap replicates to estimate Harpending's raggedness index and the sum of squared deviation (SSD) given the population expansion and spatial expansion models.

Phylogenetic trees were constructed using maximum likelihood (ML) with RAxML 8.2.12 (Stamatakis, 2014) run in the server CIPRES (Miller et al., 2010). The data matrix used was the phased haplotypes. RAxML was run with 1,000 bootstrap inferences with subsequent ML search using the gtrgamma model. The Lewis correction for ascertainment bias was implemented as it is the appropriate model for binary datasets that include only variable sites (as it is the case of SNPs) (Leache, Banbury, Felsenstein, de Oca, & Stamatakis, 2015; Lewis, 2001). Phylogenetic trees have been visualized and edited in FigTree v1.4.2.

## 3 RESULTS

### 3.1 Genetic diversity and population structure of *S. miscanthi* in China

A total of 14,520 SNPs with less than 20% of missing data per individual (0.4%–18%, 5% average) and 10% per locus (0%–9%, 2% average) were obtained for the 100 individuals from the 10 Chinese populations. Of these loci, approximately 4% deviated from the Hardy–Weinberg equilibrium (HWE) after Bonferroni correction. The levels of gene diversity (*H*_e_) observed across all populations are lower than in previous studies of *S. miscanthi* in China, but similar to that of the UK population of *S. avenae* and other cereal aphids like *Rhopalosiphum padi* (Morales‐Hojas et al., 2019) (Table 2). Overall, the Chinese population is not in HWE, and the inbreeding coefficient is positive (Table 2). The same positive, significant *F*_IS_ is observed when we group the populations into North China (Qingdao, Tai'an, Langfang, Taigu, Pingliang, and Yinchuan) and South China (Shuzou, Wuhan, Mianyang, and Kunming) following the Qinglin–Huaihe line (QHL; the traditional identified geographic north–south divide of China) (Table 2). Furthermore, several populations in China (Qingdao, Taigu, Yinchuan, and Wuhan) are also not in HWE, with significant, positive *F*_IS_ indices. Significant, positive estimates of *F*_IS_ are generally the result of inbreeding in the population or the effect of population subdivision, the Wahlund effect.

**TABLE 2:** Mean genetic diversity indices estimated for each *S. miscanthi* population, populations north and south of the QHL, and each of the identified genetic clusters (GC)

	*H*_e_	*H*_o_	*F*_IS_	*p*(random *F*_IS_ ≥ observed *F*_IS_)
Overall	0.28933	0.16720	0.3871	.0000
North	0.33122	0.19451	0.37625	.0000
Qingdao	0.3796	0.30129	0.16357	.0117
Tai'an	0.46466	0.44985	−0.01467	.5147
Langfang	0.42943	0.40584	0.00621	.4766
Taigu	0.2948	0.22396	0.21015	.0407
Pingliang	0.41717	0.34735	0.08827	.1097
Yinchuan	0.30452	0.22792	0.24868	.0344
South	0.30482	0.19169	0.33871	.0000
Shuzou	0.36834	0.313	0.10903	.0689
Wuhan	0.3134	0.19447	0.36651	.0004
Mianyang	0.46364	0.4364	−0.00632	.4827
Kunming	0.35638	0.33113	0.05435	.2473
GC1	0.46035	0.49644	−0.10550	.8083
GC2	0.40616	0.35361	0.07863	.0447
GC3	0.45541	0.42329	−0.01105	.5731
GC4	0.41227	0.37039	0.05263	.1626
GC5	0.36980	0.38917	−0.08655	.8816
GC6	0.42755	0.43997	−0.05740	.5807

Note

*H*_o_ and *H*_e_ are observed and expected (gene diversity) heterozygosity, respectively; *F*_IS_—inbreeding coefficient.

**FIGURE 2:**
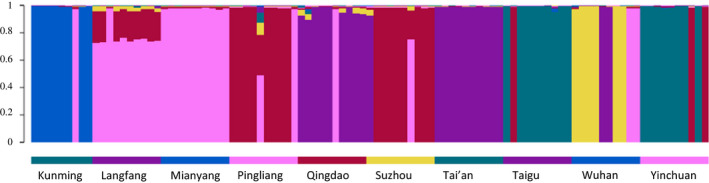
Structure analysis based on 14,520 SNPs across 10 Chinese populations, with *K* = 6. Each bar represents one individual and the colors of the bars the posterior probability that each belongs to one of the six genetic clusters. GC1—blue; GC2—magenta; GC3—yellow; GC4—green; GC5—purple; GC6—red

A first run of Bayesian clustering analyses of the population structure was carried out with five independent simulations with 100,000 burn‐in and 100,000 mcmc chains for each of *K* 1–10. Analyses of the results following the Evanno method (Evanno et al., 2005) indicated that the most likely number of clusters was *K* = 6. An additional run of five independent simulations with 100,000 burn‐in and 500,000 mcmc chains carried out for *K* 5–10 confirmed that the most likely number of *K* was 6 (Table 3, Figure 2). The structure plot shows that most sampled locations are not homogeneous, comprising individuals that are assigned to different genetic clusters (Figure 2). The genetic cluster 1 (GC1) comprises only individuals from Kunming KM1, KM2, KM3, KM4, KM5, KM6, KM7, KM9, and KM10; GC2 includes one individual from Kunming (KM8), all individuals from Langfang and Mianyang, two individuals from Pingliang (PL5 and PL10), one individual from Qingdao and Suzhou (QD6 and SZ7), and two from Wuhan (WH9 and WH10); GC3 comprises only individuals from Wuhan (WH1, WH2, WH3, WH4, WH7, and WH8); GC4 includes nine samples from Taigu (TG1, TG3, TG4, TG5, TG6, TG7, TG8, TG9, and TG10) and eight from Yinchuan (YC1, YC2, YC3, YC4, YC5, YC6, YC7, and YC9); GC5 is comprised by nine individuals from Qingdao (QD1, QD2, QD3, QD4, QD5, QD7, QD8, QD9, and QD10), all samples from Tai'an, one from Suzhou (SZ1), and two from Wuhan (WH5 and WH6); and GC6 includes eight samples from Pingliang (PL1, PL2, PL3, PL4, PL6, PL7, PL8, and PL9), eight from Suzhou (SZ2, SZ3, SZ4, SZ5, SZ6, SZ8, SZ9, and SZ10), one from Taigu (TG2), and two from Yinchuan (YC8 and YC10). These results suggest that the Wahlund effect, the mixture of genetically different populations, is the most likely reason for the significant *F*_IS_ in Qingdao, Taigu, Yinchuan, and Wuhan. This is further supported by the fact that *F*_IS_ is nonsignificant when the individuals are grouped according to the genetic cluster to which they were assigned by the Bayesian clustering analyses with Structure, and thus, genetic clusters are in HWE except GC2 (*F*_IS_ = 0.07863, *p* = .0447) that includes 27 individuals from seven different locations (Table 2).

**TABLE 3:** Table of results from Structure for the Chinese populations (a) five independent simulations for *K* 1–10, 100,000 burn‐in and 100,000 mcmc chains; (b) five independent simulations for *K* 5–10, 100,000 burn‐in and 500,000 mcmc chains

*K*	Reps	Mean Ln*P*(*K*)	Stdev Ln*P*(*K*)	Lnʹ(*K*)	|Lnʺ(*K*)|	Delta *K*
(a)						
1	5	−1,237,847.48	14.9028	NA	NA	NA
2	5	−1,068,686.16	8,702.18	169,161.32	75,998.68	8.733291
3	5	−975,523.52	35,417.94	93,162.64	25,385.46	0.716740
4	5	−856,975.42	29,834.73	118,548.10	133,411.76	4.471693
5	5	−871,839.08	51,758.53	−14,863.66	35,760.94	0.690919
6	5	−850,941.80	35,376.44	20,897.28	68,821,957.12	1,945.417782
7	5	−69,652,001.64	140,217,321.35	−68,801,059.84	137,586,196.24	0.981235
8	5	−866,865.24	73,746.76	68,785,136.40	76,730,695.74	1,040.461790
9	5	−8,812,424.58	17,564,554.27	−7,945,559.34	15,848,078.50	0.902276
10	5	−909,905.42	61,354.10	7,902,519.16	NA	NA
(b)						
5	5	−962,395	53,822.58	NA	NA	NA
6	5	−949,885	94,126.26	12,510.06	49,025,850	520.852
7	5	−5E + 07	49,016,699	−4.9E + 07	55,801,634	1.138421
8	5	−4.3E + 07	59,145,788	6,788,294	28,389,784	0.479997
9	5	−6.5E + 07	41,036,977	−2.2E + 07	59,738,205	1.455717
10	5	−2.7E + 07	23,812,471	38,136,715	NA	NA

Analysis of the molecular variance (AMOVA) indicated that the overall *F*_ST_ of *S. miscanthi* in China was 0.3254 (*p* = 0); thus, 32.54% of the total genetic variation was explained by differences between the populations. When the individuals were grouped according to their assigned genetic cluster, 40.68% of the genetic variation (*F*_CT_ = 0.4069, *p* = 0) was explained by differences among the groups (Table 4). Finally, we tested the QHL north–south subdivision of the *S. miscanthi* population previously suggested in the literature with an AMOVA. Results did not support the QHL divide hypothesis with only a nonsignificant 2.22% of the genetic variation being explained by such geographic division (*F*_CT_ = 0.0222, *p* = .2569) (Table 4).

**TABLE 4:** AMOVA of the SNP dataset from *S. miscanthi*

	Source of variation	Sum of squares	Variance components	% variation	Fixation indices
A	Among groups	146,960.625	840.81437	40.68	*F*_CT_ = 0.4068 (*p* = 0)
	Among populations within groups	23,445.277	83.52677	4.04	*F*_SC_ = 0.0681 (*p* = 0)
	Among individuals within populations	90,706.443	−22.79868	−1.10	*F*_IS_ = −0.0199 (*p* = .6556)
	Within individuals	116,543.000	1,165.43000	56.38	*F*_IT_ = 0.4362 (*p* = 0)
B	Among groups	17,982.662	43.99279	2.22	*F*_CT_ = 0.0222 (*p* = .2569)
	Among populations within groups	110,074.833	614.04835	31.01	*F*_SC_ = 0.3172 (*p* = 0)
	Among individuals within populations	133,054.850	156.47861	7.90	*F*_IS_ = −0.1184 (*p* = 0)
	Within individuals	116,543.000	1,165.43000	58.86	*F*_IT_ = 0.4114 (*p* = 0)

Note

Analyses were performed to test the following hierarchical substructure: (A) individuals grouped according to the genetic cluster assignment from Structure; (B) populations grouped according to their north–south location with respect to the Qinling–Huaihe line divide.

Pairwise *F*_ST_ tests showed that the genetic differentiation between the different populations is high and significant in most cases (Table 5). The only exceptions are between the populations of Suzhou and Pingliang, and Yinchuan and Taigu, which showed no genetic differentiation. Qingdao and Tai'an, which are separated by 300 km, showed a low but significant *F*_ST_, and Langfang, Pingliang, Mianyang, and Suzhou showed an intermediate and significant *F*_ST_, despite some of these locations being more than 1,000 km apart. The genetic differentiation between the genetic clusters is higher and significant in all pairwise comparisons (Table 6).

**TABLE 5:** Genetic differentiation between populations estimated with pairwise *F*_ST_ (below the diagonal) with significant values (*p* value < .001 after the exact test estimated with 10,100 permutations) in italics; geographic distances between samples in kilometers above the diagonal

	South populations				North populations					
	Kunming	Mianyang	Wuhan	Suzhou	Yinchuan	Pingliang	Taigu	Tai'an	Langfang	Qingdao
Kunming		750	1,287	1,872	1,,546	1,287	1,,668	1,,875	2,120	2,095
Mianyang	*0.38556*		925	1,,507	792	534	980	1,276	1,430	1,540
Wuhan	*0.35288*	*0.25497*		598	1,156	940	785	710	1,015	827
Suzhou	*0.35113*	*0.13993*	*0.20726*		1537	1,394	1,010	666	980	530
Yinchuan	*0.47602*	*0.36352*	*0.48536*	*0.31532*		348	564	990	934	1,279
Pingliang	*0.36378*	*0.12852*	*0.22950*	−0.00006	*0.31710*		550	947	980	1,242
Taigu	*0.52138*	*0.43957*	*0.54790*	*0.39557*	−0.01011	*0.40377*		426	450	704
Tai'an	*0.40604*	*0.37101*	*0.23802*	*0.29632*	*0.46615*	*0.33196*	*0.52301*		390	300
Langfang	*0.37918*	*0.13704*	*0.26178*	*0.11658*	*0.34895*	*0.12602*	*0.42251*	*0.35654*		508
Qingdao	*0.34611*	*0.27895*	*0.17379*	*0.20910*	*0.40618*	*0.24716*	*0.46340*	*0.05756*	*0.27520*	

**TABLE 6:** Genetic differentiation (pairwise *F*_ST_) between the six genetic clusters (GC) identified with Structure

	GC 1	GC 2	GC 3	GC 4	GC 5	GC 6
Genetic cluster 1	0.00000					
Genetic cluster 2	0.42822	0.00000				
Genetic cluster 3	0.46296	0.13319	0.00000			
Genetic cluster 4	0.43198	0.30765	0.32792	0.00000		
Genetic cluster 5	0.65910	0.45260	0.51381	0.54811	0.00000	
Genetic cluster 6	0.55366	0.40099	0.44521	0.38811	0.79219	0.00000

Note

All pairwise comparisons were significant (*p* = 0) as estimated with 10,100 permutations. GC 1 includes only individuals (*n* = 9) from Kunming; GC 2 comprises individuals from Kunming (1), Langfang (10), Mianyang (10), Pingliang (2), Qingdao (1), Suzhou (1), and Wuhan (2); GC 3 consists of individuals from Wuhan (6); GC 4 includes individuals collected in Taigu (9) and Yinchuan (8); GC 5 is formed by individuals from Qingdao (9), Suzhou (1), Tai'an (10), and Wuhan (2); and GC 6 includes individuals from Pingliang (8), Suzhou (8), Taigu (1), and Yinchuan (2).

The Mantel test was nonsignificant when no genetic structure within *S. miscanthi* is considered (slope 0.000044, *R* = 0.1514, *p* = .205) rejecting the isolation by distance (IBD) hypothesis. However, as the Bayesian clustering analyses showed, there are 6 likely genetic clusters and this subdivision of the population can bias the test. To control for this, we performed Mantel tests for each of the identified clusters 2, 5, and 6 separately (these clusters included individuals from more than one location) but results were still nonsignificant (GC 2: slope −0.000022, *R* = −0.1496, *p* = .719; GC5: slope −0.000016, *R* = −0.0387, *p* = .538; GC6: slope 0.000036, *R* = 0.7177, *p* = .118). Nevertheless, the low number of individuals for some of the locations within each cluster limits the statistical power of the tests.

**FIGURE 3:**
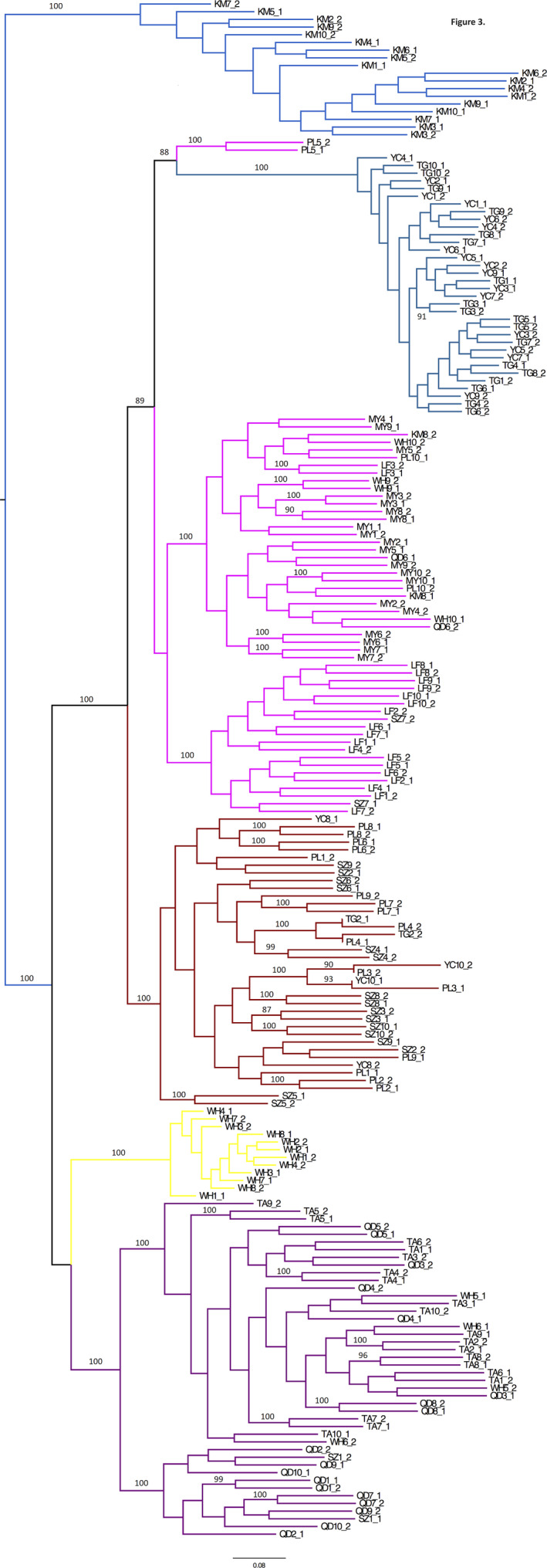
Midpoint rooted phylogenetic tree estimated with RAxML for the *S. miscanthi* phased haplotypes from China using a dataset of 14,520 SNPs. The six genetic clusters are highlighted with different colors, corresponding to the colors in the barplot of Figure 2. Labels on branches are bootstrap values >90%. GC1—blue; GC2—magenta; GC3—yellow; GC4—green; GC5—purple; GC6—red

Phylogenetic analysis of *S. miscanthi* SNP dataset resulted in a phylogeny that reflected the inferred population structure (Figure 3). The evolutionary tree shows six main clades with high bootstrap support corresponding to the identified genetic clusters. The relationships between the clades are also well supported, and they provide further information regarding their evolutionary relationship. Thus, the Kunming population (Genetic Cluster 1) is the sister clade to a supergroup comprising the other clades. Haplotypes from Tai'an and Qingdao (GC5), which are geographically closer to each other than to other locations, cluster together with 4 haplotypes from two individuals collected in Wuhan and two haplotypes from Suzhou; the sister group to this clade comprises only samples from Wuhan (GC3), despite being geographically distant. The sister clade to this clade comprising these two clusters includes the genetic clusters 2, 4, and 6; within this clade, GC6 comprising individuals from Yinchuan, Pingliang, Suzhou, and Taigu is the sister group to GC4 and GC2, which include individuals from most geographic locations. Overall, there is no geographic signature observed in the phylogenetic tree in accordance with the population structure analyses, except for a clustering of the majority of the individuals from Kunming in a separate clade.

### 3.2 Genetic diversity and population structure of *S. avenae* in the UK

A total of 846 SNPs with less than 25% of missing data per locus (0%–24%, 13% mean) and <50% per individual (2%–48%, 14% mean) were identified in 98 individuals from 12 sampling locations across England. Approximately 19% of these loci are deviated from the HWE after Bonferroni correction. The gene diversity, estimated as the He, is high across all populations (Table 7), although it is lower than the He observed in a previous analysis of the UK populations using four microsatellites (Llewellyn et al., 2003), although comparison of the results obtained with genome‐wide markers obtained in the present analysis and that of microsatellites has to be done with care. The *H*_o_ was higher in all populations compared to the *H*_e_, resulting in high negative *F*_IS_ estimates which indicate that the *S. avenae* in England is not in HWE (Table 7). This contrasts with the situation of the UK population 15–20 years ago where most markers in the analyzed populations were in HWE (Llewellyn et al., 2003). Although the negative *F*_IS_ values were not significant according to the permutation test performed by Arlequin 3.5, it should be noted that this test evaluates the probability of obtaining random values that are higher than the observed ones rather than the probability of random values being more negative. Therefore, it is most probable that the *p* values reported are not correct for these values. When Fis were estimated using Genepop, all were also negative and most were significant using the exact test. The negative *F*_IS_ is the result of an excess of heterozygotes, and it is considered a signature of clonal reproduction.

**TABLE 7:** Mean genetic diversity indices estimated for each *S. avenae* population in England and overall

	*N*	*H*_e_	*H*_o_	*F*_IS_ (Arlequin)	*F*_IS_ (Genepop)
Overall	196	0.28433	0.41243	−0.6928	
Broom's Barn	20	0.37289	0.54525	−0.6631	−0.5101**
Hereford	20	0.37214	0.53194	−0.8069	−0.4745**
Kirton	18	0.38078	0.54714	−0.7750	−0.4908*
Newcastle	20	0.38088	0.56614	−0.7302	−0.5401**
Preston	12	0.40722	0.57489	−0.7154	−0.4879
Rothamsted	16	0.37388	0.51535	−0.6035	−0.4271
Silwood Park	14	0.39285	0.54213	−0.7222	−0.4382
Starcross	2	–	–	–	–
Wye	18	0.36667	0.51283	−0.7137	−0.4460
Wellesbourne	20	0.35591	0.50472	−0.6003	−0.4594**
Writtle	18	0.36290	0.51392	−0.6405	−0.4660**
York	18	0.37310	0.53559	−0.7419	−0.4913**

Note

*N* is the number of gene copies (2 × number of individuals); *H*_o_ and *H*_e_ are observed and expected (gene diversity) heterozygosity, respectively; *F*_IS_—inbreeding coefficient. Starcross values are not included as it was represented by one single individual.

**FIGURE 4:**
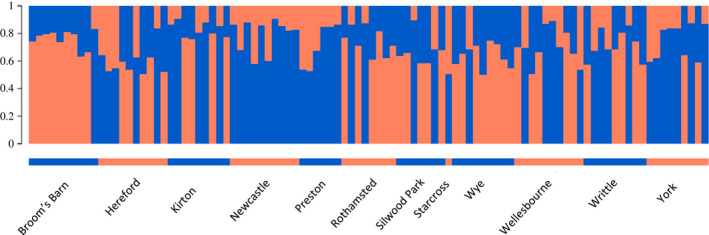
Structure analysis based on 846 SNPs across 12 English populations of *S. avenae* for *K* = 2. Each bar represents one individual and the colors of the bars the posterior probability that each belongs to each of the genetic clusters

Bayesian clustering analysis was run with 5 replicates of 500,000 burn‐in and 1,000,000 mcmc chains to ensure convergence. Results were analyzed following the Evanno method (Evanno et al., 2005), which suggested that the most likely number of genetic clusters is *K* = 2 (Table 8). However, this method does not estimate the deltaK for *K* = 1 and the mean LnP(K) is maximized for *K* = 1 suggesting that the most likely number of clusters is 1 (Table 8). In addition, the standard deviation increases from *K* = 2, which usually happens after reaching the best *K*. Finally, there is no clear distribution of individuals into 2 differentiated clusters in the bar plot resulting from the analyses with *K* = 2, with all of them having some probability of belonging to each cluster (Figure 4). An additional nonmodel‐based analysis to verify the number of clusters identified by Structure in the data was performed with an unsupervised clustering approach (DAPC) (Jombart et al., 2010). For this, the “find.clusters” function of the R package adegenet 2.1.2 was used (Jombart, 2008; Jombart & Ahmed, 2011). This function applies a DAPC method to identify the optimal number of clusters using a model selection criterion, in this case the Bayesian information criterion (BIC). The BIC is minimized when *K* = 3 (BIC = 384.6787) but the difference with *K* = 1 (BIC = 386.0667) is of 1.388 and differences smaller than 2 between two models are considered to be weak evidence (Raftery, 1995). When automatic selection was applied using the good fit criterion, the number of optimal clusters retained was *K* = 1. Taking all the results in consideration, they suggest that there is no population structure but just one genetic cluster.

**TABLE 8:** Table of results from Structure for the English populations (five independent simulations for *K* 1–12, 500,000 burn‐in and 1,000,000 mcmc chains)

*K*	Reps	Mean Ln*P*(*K*)	Stdev Ln*P*(*K*)	Lnʹ(*K*)	|Lnʺ(*K*)|	Delta *K*
1	5	−55,651.5	0.8927	NA	NA	NA
2	5	−59,437.7	795.9415	−3,786.2	4,110.34	5.164123
3	5	−67,334.3	1,512.473	−7,896.54	4,447.6	2.940614
4	5	−70,783.2	4,688.426	−3,448.94	3,568.64	0.761159
5	5	−70,663.5	3,032.39	119.7	6,005.16	1.980339
6	5	−76,549	5,716.411	−5,885.46	12,612.16	2.206308
7	5	−69,822.3	2,700.041	6,726.7	9,361.38	3.467125
8	5	−72,456.9	7,835.535	−2,634.68	7,552.04	0.963819
9	5	−67,539.6	3,157.942	4,917.36	4,039.52	1.279162
10	5	−6,6661.7	4,041.049	877.84	2,479.22	0.613509
11	5	−63,304.7	2,208.609	3,357.06	5,218.48	2.362791
12	5	−65,166.1	3,124.338	−1,861.42	NA	NA

Analyses of the molecular variance (AMOVA) also supported the existence of one single gene cluster. The overall diversity of *S. avenae* in England was low *F*_ST_ = 0.001 and nonsignificant among populations, indicating a low differentiation level. When the individuals were clustered according to the Structure results for *K* = 2, assigning individuals to each genetic cluster according to the membership coefficient estimated with clump, the amount of genetic variation that was explained between groups was 2.33% (*p* = 0) (Table 9). These results support those of Structure with one single genetic cluster and no population structure. In addition, the genetic differentiation estimates (*F*_ST_) were all negative (Table 10), which indicates that there is no divergence between the different locations. Similarly, when the two possible genetic clusters identified with Structure are compared, the *F*_ST_ is low (0.0158; *p* = 0), further supporting the lack of population structure in *S. avenae* from England. Mantel test rejected the IBD hypothesis (slope 0, *R* = n.a., *p* = 1) as expected given the lack of genetic differentiation between locations (*F*_ST_).

**TABLE 9:** AMOVA of the SNP dataset from *S. avenae*

	Source of variations	Sum of squares	Variance components	% variation	Fixation indices
A	Among groups	203.571	1.87014	2.33	*F*_CT_ = 0.0233 (*p* = 0)
	Among populations within groups	397.908	−0.21891	−0.27	*F*_SC_ = −0.0028 (*p* = .8146)
	Among individuals within populations	1,765.164	−55.77004	−69.41	*F*_IS_ = −0.7087 (*p* = 1)
	Within individuals	13,177.500	134.46429	167.36	*F*_IT_ = −0.6736 (*p* = 1)

Note

Analyses were performed to test the genetic cluster assignment from Structure, *K* = 2.

**TABLE 10:** Genetic differentiation between populations estimated with pairwise *F*_ST_ with significant values (*p* value < .001 after the exact test estimated with 10,100 permutations) in italics; geographic distances between samples in kilometers above the diagonal

	BB	H	K	*N*	P	RT	SP	SX	W	We	Wr	Y
BB	–	217	79	358	286	80	124	335	124	148	59	222	
H	−0.0195	–	197	350	195	160	155	176	268	71	215	240
K	−0.0311	−0.0297	–	275	206	121	170	347	205	130	136	138
N	−0.0051	−0.0244	−0.0438	–	161	384	425	524	480	331	410	137
P	−0.0206	−0.0454	−0.0590	−0.0499	–	277	304	363	387	195	319	119
RT	−0.0321	−0.0410	−0.0466	−0.0281	−0.0433	–	50	253	111	97	54	250
SP	−0.0292	−0.0471	−0.0419	−0.0374	−0.0528	−0.0549	–	213	112	113	83	294
SX	−0.4919	−0.4619	−0.4359	−0.5208	−0.5768	−0.5711	−0.4966	–	314	220	298	415
W	−0.0329	−0.0328	−0.0420	−0.0269	−0.0430	−0.0532	−0.0470	−0.4958	–	208	71	340
We	−0.0162	−0.0460	−0.0461	−0.0301	−0.0447	−0.0408	−0.0515	−0.5804	−0.0427	–	150	205
Wr	−0.0243	−0.0420	−0.0501	−0.0362	−0.0483	−0.0454	−0.0507	−0.5559	−0.0451	−0.0395	–	272
Y	−0.0139	−0.0273	−0.0462	−0.0449	−0.0515	−0.0405	−0.0444	−0.5112	−0.0254	−0.0406	−0.0406	–

Abbreviations: BB, Broon's Barn; H, Hereford; K, Kirton; N, Newcastle; P, Preston; RT, Rothamsted; SP, Silwood Park; SX, Starcross; W, Wye; We, Wellesbourne; Wr, Writtle; Y, York.

**FIGURE 5:**
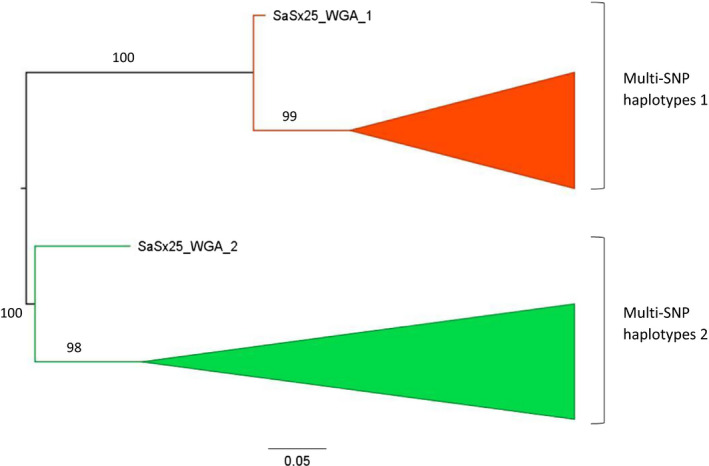
Midpoint rooted phylogenetic tree estimated with RAxML for the *S. avenae* phased haplotypes from England using a dataset of 846 SNPs. The two‐phased multimarker haplotypes from every individual are colored in red and green, and the clades have been collapsed except for the earliest branching haplotype of each clade. Labels on branches are bootstrap values >90%

Phylogenetic analyses were run using the two multi‐SNP haplotypes of each individual. The inferred ML phylogeny comprised two major clades highly supported by bootstrap (Figure 5). Each of these two clades included one of the multi‐SNP haplotypes from each individual, so that each multi‐SNP haplotype is more closely related to a haplotype from another individual than to the second haplotype from the same individual. This type of phylogenetic topology can be the result of asexual reproduction from a single individual, in which all copies from each of the extant haplotypes derive from one common ancestral haplotype with no recombination. The basal node would represent the common ancestral asexual individual. Thus, the phylogeny supports the lack of population structure and suggests that there is one single clone or genetic cluster dominating the English population of *S. avenae*, and the asexual reproduction of this lineage.

Analyses of the historical demography of this species in England showed a population and spatial expansion of the genetic cluster identified in the population genetic structure analysis, which would be consistent with the increase in frequency of a single insecticide‐resistant clone in the population and its spread across different locations. Thus, Fu's *F*_s_ index was negative (*F*_s_ = −25.41, *p* = 0), which is a signature of population expansions. In addition, the mismatch analyses showed a unimodal distribution and failed to reject departure from the expansion models, resulting in a nonsignificant Harpending's raggedness index (0.0001, *p* = 1) for the demographic and spatial expansion models, and nonsignificant sum of squared deviation (SSD) for the spatial expansion model (*p* = .438) while significant at the 5% for the population expansion model (*p* = .031).

## 4 DISCUSSION

The results from the present study provide information about the evolution of two closely related species of cereal aphids under different environments and agricultural landscapes. The study demonstrates that the populations of *S. miscanthi* and *S. avenae* in China and England have evolved in the last 5 to 20 years, which could be as a result of environmental and human‐induced changes such as insecticide use, and the genetic structure and diversity have changed in comparison with that observed in earlier studies. This contrasts with what it has been observed previously in another cereal aphid in England, *Rhopalosiphum padi*, whose population has not shown any change in genetic diversity or structure at least since 2003 (Morales‐Hojas et al., 2019).

Results of the study indicate that *S. miscanthi* in China has a higher diversity than previously identified (Guo et al., 2005; Wang et al., 2016). This can be explained by the higher number of genome‐wide molecular markers that have been used in the present analysis in comparison with the five microsatellites of previous studies, but the estimated levels of genetic differentiation were similar to those observed in *S. avenae* in China using the same five microsatellites (Xin, Shang, Desneux, & Gao, 2014). However, *S. avenae* is known to be present only in Yili, Xinjiang region in the northwest (Zhang, 1999), and the two *Sitobion* species are morphologically very similar (Choe, Lee, & Lee, 2006; Hales, Foottit, & Maw, 2010), so it is likely that samples from China have been identified as *S. avenae* in some studies and as *S. miscanthi* in others (Blackman & Eastop, 2017). In addition, it could also be the case that there is undescribed taxonomic diversity within *S. miscanthi* in China, and the high levels of genetic differentiation observed could be explained by unidentified races. This is the case in Australia, where there are at least three chromosomal races (Hales, Chapman, Lardner, Cowen, & Turak, 1990), so it is possible that the situation in China is complex as well. Indeed, the high genetic differentiation observed in the present analysis between the six genetic clusters identified suggests that there is at least the same number of different taxonomic units, though the present study is not capable to determine whether they represent subspecies, host races, or chromosomal races as in Australia and New Zealand. Also, it is interesting to note that the individuals show little genetic admixture except for samples from Langfang, one sample from Suzhou and one from Pingliang, which have a proportion of SNPs from the GC2 and GC6. This could be the result of a lack of reproduction between genetic clusters, despite individuals from different clusters coexisting in the same geographic locations.

While *S. miscanthi* in Australia and New Zealand is predominantly functional parthenogenetic (Sunnucks et al., 1996; Wilson et al., 1999), the present study indicates that the populations in China show either a heterozygote deficiency (significant, positive *F*_IS_) or are in HWE (not significant *F*_IS_), suggesting that the species reproduces predominantly by cyclical parthenogenesis. The significant deficiency of heterozygosity in populations can be explained by the presence of different genetic clusters (Wahlund effect) observed in several populations, and the *F*_IS_ is not significant for the genetic clusters indicating that they are in HWE (with the exception of the GC2). This contrasts with previous studies that inferred an excess of heterozygotes in the northern populations, characteristic of anholocyclic lineages, while in the southern populations there was a deficiency of heterozygosity, which is observed in inbred sexual populations or the result of admixed populations (Wahlund effect) (Wang et al., 2016). However, it is usually the case that in colder regions populations are cyclical parthenogenetic as the aphids undergo a phase of sexual reproduction to produce eggs to overwinter; on the other hand, southern populations in warmer regimes can survive as parthenogenetic individuals throughout the year. The results of Wang et al. (2016) indicate that the contrary would be the case in Chinese *S. miscanthi*, which is unexpected. Another previous study of *S. miscanthi* in China showed that northern populations had heterozygote deficiency while the southern populations were in HWE or had an excess of heterozygotes, although the significance was not tested (Guo et al., 2005). The contrasting results between the present and previous analyses are also at the population subdivision. Thus, we observe no significant north–south differentiation at the QHL traditional geographic division of the country, identified in one previous study (Wang et al., 2016), and there is no evidence for isolation by distance (Guo et al., 2005). The population structure identified in the present study and the phylogenetic analysis suggests that there are six highly differentiated genetic clusters, and admixture results suggest that there is little genetic exchange between them. These clusters do not correspond to geographic regions, as observed also in the phylogeny, and individuals from populations geographically separated by long distances can belong to the same genetic cluster. GC1 comprising individuals from only Kunming and GC3 with individuals only from Wuhan are geographically restricted to one location; the Kunming population is more geographically distant to the other locations, which could explain its genetic isolation, although one individual sampled in Kunming shows a GC2 genotype. This suggests that there is long‐distance dispersal of *S. miscanthi* aphids across China, although the high differentiation observed between the genetic clusters and the low genetic admixture in individuals suggest low interbreeding between them.

In England, the level of genetic differentiation is low across the different populations of *S. avenae* sampled and there is no evidence for genetic structure. This is in accordance with what it was previously observed (Llewellyn et al., 2003). This population homogeneity was taken to be the result of long‐distance dispersal of aphids, and results from the present study corroborate this. The population of *S. avenae* in England, however, has evolved in the last 15 years. While most of the markers and populations studied in 1997–1998 were in HWE, and the population showed an increase in cyclical parthenogenetic proportion with latitude (Llewellyn et al., 2003), this study shows that the present English population has an excess of heterozygotes and indicates strong clonality. This suggests that anholocycly is predominant across its range, and there is no evidence for cyclical parthenogenesis occurring toward the north of the country as expected. This change in the *S. avenae* population could be the result of insecticide resistance evolution. In 2011, a knockdown resistance (*kdr*) mutation to pyrethroids was detected in England's population of *S. avenae*, and studies showed that the clone that gained this mutation spread and increased its proportion in the population from 2009 to 2014, and was also observed in Ireland from 2013 (Dewar & Foster, 2017; Foster et al., 2014; Malloch et al., 2014, 2016; Walsh et al., 2019). It is therefore likely that this pyrethroid‐resistant clone, which is a facultative parthenogenetic clone (Walsh et al., 2019), has continued to spread and increase in proportion, being now dominant in the English population. The single genetic cluster identified by the Bayesian and AMOVA analyses in this study could correspond to this clone. This is supported by the phylogenetic analysis, which shows a topology that can be explained by asexuality of the *S. avenae* population. Also, the levels of gene diversity (as measured by the He) are now lower than those observed in 2003 in the panel of four microsatellites (Llewellyn et al., 2003). In addition, analyses of the demographic history of the population in England indicate that there has been a population demographic and spatial expansion. Thus, as one clone gained the resistance to pyrethroids in a given location, it increased in number in the location but also expanded its distribution as it spread to other regions via migration. However, further analyses will need to be carried out to demonstrate this.

Overall, this study shows that the populations of two species of cereal aphids of the genus *Sitobion* have evolved in recent years in two geographically distant regions under different environmental and human‐influenced conditions. The diversity of *S. miscanthi* in China needs to be investigated more comprehensively, as the high level of genetic differentiation suggests the existence of yet unidentified forms. In contrast, the diversity of *S. avenae* has been affected by the evolution of pyrethroid resistance as shown in previous studies, and a single genetic cluster is now dominating the English population as shown in this present study. Although it is possible that the identified genetic cluster corresponds to the insecticide resistance clone, further analyses are needed to demonstrate this. In contrast to this, *S. miscanthi* has not gained insecticide resistance despite having been subject also to its use. In England, the bird cherry—oat aphid, *R. padi*, has not evolved resistance to insecticides either, despite being sympatric with *S. avenae* and therefore subject to the same agricultural practices. Why some species evolve resistance while other do not it is still a matter of study. It has been shown in *Drosophila melanogaster* that thermotolerance influences the development and spread of insecticide resistance (Fournier‐Level et al., 2019). Similarly, the distribution of cyclical and obligate parthenogenetic aphids is strongly influenced by temperature, so it is possible that there is an indirect relationship between life‐cycle type and insecticide resistance evolution in aphids. Further studies in aphids would need to be carried out to test whether there is a relationship between thermotolerance, life‐cycle types, and the evolution of the *kdr* and super‐*kdr* mutations; for this, the population of *S. avenae* in England, where it is predominantly anholocyclic, its sympatric population *R. padi* and the related species *S. miscanthi* in China, which are predominantly cyclical parthenogenetic, can be a useful system to test the hypothesis.

## CONFLICT OF INTEREST

The authors declare that there is no conflict of interest.

## AUTHOR CONTRIBUTION

**Ramiro Morales‐Hojas:** Conceptualization (lead); Data curation (lead); Formal analysis (lead); Funding acquisition (lead); Investigation (lead); Methodology (lead); Project administration (lead). **Jingxuan Sun:** Data curation (supporting); Formal analysis (supporting); Investigation (supporting); Methodology (equal). **Fernando Alvira Iraizoz:** Data curation (supporting); Formal analysis (supporting); Investigation (supporting); Methodology (supporting); Writing‐original draft (supporting). **Xiaoling Tan:** Data curation (supporting); Investigation (supporting); Methodology (supporting). **Julian Chen:** Conceptualization (equal); Data curation (supporting); Formal analysis (supporting); Funding acquisition (equal); Investigation (supporting); Methodology (supporting); Project administration (equal).

## Data Availability

All the DNA sequencing reads have been uploaded to the European Nucleotide Archive (ENA) and can be found under the study with accession number PRJEB36151. Accession numbers for the samples’ sequencing reads are ERR3810098–ERR3810316. Vcf files resulting from the FreeBayes and the filtered vcf files, fasta alignments of the SNPs used in the phylogenetic analyses, and the phylogenetic trees in newick format have been deposited in Dryad (https://doi.org/10.5061/dryad.k0p2ngf5x).
